# Avian Influenza H5N1 Infection During Pregnancy: Preparing for the Next Flu Pandemic and Improving Perinatal Outcomes

**DOI:** 10.3390/v18020212

**Published:** 2026-02-06

**Authors:** Matthew J. Zuber, Callie L. Brown, Cara B. Janusz

**Affiliations:** 1 Department of Obstetrics and Gynecology, Section of Maternal-Fetal Medicine, Wake Forest University School of Medicine, Winston-Salem, NC 27157, USA; 2 Center for Vaccines at the Extremes of Aging (CVEA), Wake Forest University School of Medicine, Winston-Salem, NC 27157, USA; callie.l.brown@wfusm.edu (C.L.B.);; 3 Department of Pediatrics, Wake Forest University School of Medicine, Winston-Salem, NC 27157, USA; 4 Department of Implementation Science, Wake Forest University School of Medicine, Winston-Salem, NC 27157, USA

**Keywords:** influenza during pregnancy, avian flu, H5N1, flu and perinatal outcomes

## Abstract

Influenza (flu) is a common respiratory virus with seasonal global spread. Zoonotic viruses can occasionally cross species, leading to pandemic-level spread, and for flu viruses, this is considered an “antigenic shift”. The flu can be particularly severe during pregnancy due to immune system adaptations that occur during pregnancy, with prior global pandemics causing excess hospitalizations, deaths, and other complications in the mothers and the neonates. We aim to review the current literature with respect to novel avian H5N1 and the potential impact of infection with flu during pregnancy. A systematic literature search was conducted. Here we provide a rapid summary of epidemiology and understanding of viral spread, published risks of H5N1 in pregnancy, the unique physiologic, cellular, and molecular adaptations making H5N1 infection unique in pregnancy, implementation of an effective vaccine program in event of a pandemic specific to pregnant individuals, optimizing peripartum care for infected individuals, and direction for future research to direct vaccine strategy and mitigate risks in a future flu pandemic.

## 1. Introduction

Influenza (flu) is a common respiratory virus, and it has long been a part of the human story of survival and evolutionary adaptation of the immune system. Every year, the virus maintains global spread and certain strains become the most dominant, circulating seasonally and often causing respiratory or generalized symptoms in all age groups; often, children and the elderly are at the greatest risk of severe disease. Under many circumstances, patients survive the infection with nothing more than bothersome symptomatology, but the virus has the capacity to be devastating to the point of requiring hospitalization and, in some cases, resulting in death. Additionally, the virus may harbor among asymptomatic people, with household transmission studies suggesting a pooled estimate of 16% asymptomatic individuals, though this estimate varies widely by strain and study design [1]. The flu virus has a knack for adaptation and spreads when reassortment or zoonotic leaps occur. Humans develop both humoral and cellular immune responses to viral components.

Pregnancy has long been recognized as a risk factor for severe disease. Historically, pregnant persons have suffered increased rates of morbidity and mortality compared to their non-pregnant peers during the last several flu A pandemics of the past century, most recently during the 2009 H1N1 pandemic [2]. Similarly, pregnant persons had elevated morbidity and mortality during the recent COVID-19 pandemic [3]. Vaccine clinical trials continue to routinely exclude pregnant persons, creating substantial limitations in closing equitable care gaps for pregnancy and generating robust and early data during a pandemic to help support vaccine programs for pregnant persons at heightened risk for severe disease.

Avian influenza (AIV) subtype H5N1 has been a virus of recent concern, given documented cross-species transfer to humans [4] and in recognized cases, the infections have been known to have exceedingly high mortality or severe morbidity rates, prompting fear that in the event of a mutation allowing human-to-human transmission and widespread infection, this virus could lead to a devastating human pandemic. Very little is known about the risks of H5N1 in pregnancy, and even less about the potential strategies to mitigate risk without an effective vaccination strategy or to treat and/or provide prophylaxis for the virus, beyond what is already known about antivirals utilized for currently circulating flu strains.

In this review, we aim to discuss H5N1 infection in pregnancy and to propose a framework to approach pregnancy and perinatal management for a future AIV pandemic. Our team conducted a literature search using Medline Ovid through the Wake Forest University School of Medicine Carpenter Library and identified articles of interest describing considerations of H5N1 infection and pregnancy. We review what is currently understood, discuss the considerations for antepartum, intrapartum, and postpartum care of mothers with infections, care of their neonates, and provide context for future research and vaccine development, and vaccination strategies specific to pregnant women.

## 2. Epidemiology of H5N1 in Pregnancy and Future Pandemic Risk

Flu A subtypes are named for the hemagglutinin surface glycoprotein (H) and the neuraminidase (N) components. Influenza A viruses have global pandemic potential when there is an antigenic shift with high virulence and potential for transmission [5]. Reassortment of strains may occur due to gene sharing of H and N types when there is coinfection in the same animal, with a wide range of H and N combinations globally. Circulating seasonal strains undergo seasonal evolution to the “head” region of the hemagglutinin (HA) viral capsule surface glycoprotein. However, currently available flu vaccines for clinical use target this variable “head” region, as it is the antigenically dominant response in humans [6]. An active international goal is the research and development of a more “universal” flu vaccine that generates a durable response by creating memory immunity to preserved antigens shared across many subtypes of influenza. One viable candidate antigenic target for vaccine development is the HA stem, which is a more phylogenetically preserved, with promising pre-clinical data supporting this approach [7,8].

Avian flu (AIV) has a reservoir in wild birds with release of virus through feces and respiratory routes, but its spread to other mammals has occurred in recent years, including confirmed cases in dairy cattle [9]. Human infections have often been reported secondary to direct contact with known infected birds or domesticated animals [4]. Known potentially highly pathogenic AIV types include H5N1, H7N9, and H5N1 [10,11]. While reports of mammalian infections have increased, human cases have thus far remained rare. According to the United States Centers for Disease Control and Prevention (CDC), sporadic cases continue to be reported in the US among poultry and dairy cattle workers. As of December 2025, there have been no reported cases of person-to-person spread and only 2 deaths among 71 reported cases since streamlined reporting was introduced in 2024 [4]. Cases have presented with typical respiratory symptoms like those caused by circulating flu strains, but also with gastrointestinal (GI) symptoms, due to the potential to infect the gastrointestinal tract [12].

A systematic review from Purcell et al. in 2025 identified only 30 documented cases of AIV in pregnancy in the published literature, which included 16 cases of H5N1 among other flu subtypes [10]. Among all reported cases associated with pregnancies, there was an exceedingly high case-fatality rate observed, with over 90% risk of maternal death and 87% risk of perinatal death. Cases occurred in China, Vietnam, Egypt, and Indonesia. Of the 30 pregnancy-associated cases reported, there were no discernible patterns in the risk of infections or outcomes between trimesters of pregnancy. Even when accounting for reporting bias, the lethality observed in these few cases is starkly concerning when compared to public health impacts observed during previous flu pandemics over the past century (Spanish Flu 1918 and H1N1 California 2009), which reported increased odds of morbidity or mortality for infections during pregnancy [13,14].

## 3. Physiologic Adaptations of H5N1 in Pregnancy and Perinatal Outcomes

Respiratory physiological adaptations and immune adaptations both contribute to an increased risk of viral infection during a pregnancy. Specifically, an increased minute ventilation, decreased functional residual capacity, decreased total lung capacity, and increased bronchial and upper airway epithelial edema in response to estrogen and progestins contribute to worse outcomes with severe respiratory illnesses [15]. Additionally, immune adaptations in pregnancy include an imbalance toward humoral/B cell activity and overall decreased T cell function, including a shift toward Th2 helper and away from interferon-γ, both of which may impact susceptibility to certain viral infections; however, these impacts are pathogen-specific [16]. In vitro and animal studies have suggested unique infectious potential for H5N1 and raise the question of the possibility of vertical transmission [11,17]. Typical flu strains have low levels of viremia, and the risk of vertical/perinatal transmission is rare. A BALB/c mouse model was used to show that after midtrimester intranasal inoculation with the H5N1 strain, at animal sacrifice five days post infection, there were detectably high viral loads within the placental and fetal tissues, suggestive of potential for vertical transmission [18]. An in vitro study of mimicked human syncytiotrophoblast cells, an important barrier in the hemochorial placental unit of the human, showed that these cell types could be infected with H5N1, while in vivo studies in a mouse model with H1N1 suggests disruption of the syncytiotrophoblast cellular placental barrier which may correlate with the placentally mediated adverse pregnancy outcomes seen with severe flu infections, such as fetal growth issues or preterm birth [19].

**Figure 1:**
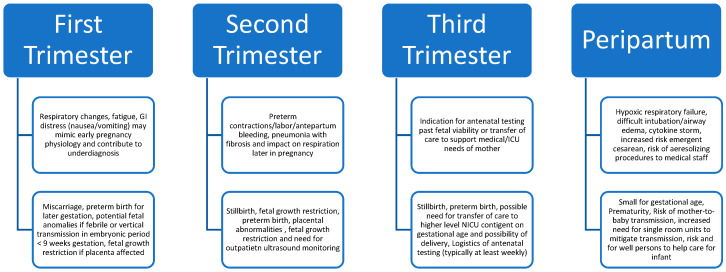
Potential maternal and fetal/neonatal impacts of avian influenza in pregnancy. The chart is organized by gestational time frame of infection to spur consideration of the timing of infection and the risks to consider with infection, depending on gestational age, though this does not mean each risk is entirely limited to that trimester, as impacts may ultimately be broad across all trimesters and peripartum.

In humans, severe flu infection often presents with severe lymphocytic lung infiltration and an associated inflammatory milieu leading to inflammation-driven lung damage; uniquely with H5N1, inflammatory activation may drive lung-induced fibrosis, raising a potential future target for therapeutic options [20]. Similar trends of immune “overactivation” were seen with the COVID-19 pandemic [21]. In a study of human-derived monocyte-derived macrophages, in vitro infection with AIV H5N1 led to higher expression of pro-inflammatory cytokines CCL2, CCL3, CCL5, and CXCL10 when compared to infection with circulating human strains H1N1 [22]. In a post-mortem analysis of a pregnant woman who died nine days after the onset of symptoms after severe H5N1 infection at four months of gestation, in situ hybridization revealed cellular infection of the cytotrophoblast (placental) as well as resident immune cells within the placenta called Hofbauer cells. Additionally, fetal bronchi, alveolar pneumocytes, and circulating mononuclear cells were infected with H5N1, providing evidence of transplacental vertical infection in humans [12]. Based on what is known about H5N1 pathogenesis and previous flu pandemics, we outline in Figure 1 a proposed list of specific maternal and fetal/neonatal considerations arranged by period of exposure during gestation.

Little has been published regarding perinatal outcomes in peripartum H5N1 infections. Based on case reports and series of cases over the last few decades, there is a suggestion of increased fetal risk of spontaneous abortion, spontaneous preterm birth, fetal growth restriction/small for gestational age, and perinatal death [10,11]. Maternal risks include peripartum death, with a trend of infections detected toward the mid to late gestational period [10,11]. Prior history with the H1N1 pandemic would suggest increased risk of sepsis, hospitalization, and intensive care unit needs, including in the peripartum period [13,14].

## 4. Considerations for Pregnancy Management in the Event of H5N1 Pandemic

**Figure 2:**
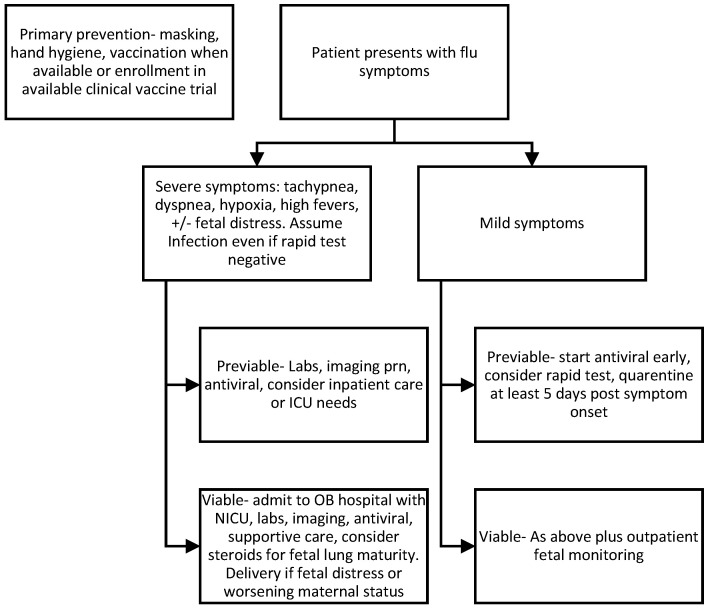
Proposed initial clinical algorithm for H5N1 in pregnancy in the event of a global pandemic.

We propose a basic algorithm for initial clinical management of suspected H5N1 in pregnancy (Figure 2). Initial care should be guided by the severity of disease presentation, with early consideration for maintaining a safe airway, adequate oxygenation and ventilation, and stabilization of the mother prior to fetal assessment in the event of critical illness. Imaging studies should be based on clinical judgment of necessity, accounting for a balanced approach to reducing radiation exposures when feasible [23]. Pregnant patients should wear masks when reasonable to do so to reduce transmission for themselves and to protect clinical staff, family members, and public contacts. While higher quality respirators (such as N95^®^) likely offer improved protection from small viral particles over traditional surgical single-use masks, pragmatic clinical trials have shown similar protection for healthcare workers using traditional surgical masks compared to N95^®^ respirators during non-aerosol generating medical care [24,25]. At the outset, during the acute infectious window, remote monitoring of vital signs or virtual care options may be considered to limit exposures, and home vitals and fetal assessment have a reasonable correlation with in-clinic assessments [26]. For those not requiring urgent delivery or indicated premature birth due to severe disease, heightened outpatient monitoring is advisable until pathogen-specific data can be established. Perinatal outcomes of interest during other respiratory viral pandemics have included fetal growth restriction (FGR)/small for gestational age (SGA), miscarriage, fetal anomalies with severe first-trimester infection, spontaneous preterm birth, and stillbirth [10,15]. There are well-established screening protocols that vary by institution to detect growth restriction by ultrasound (typically monthly sonography to evaluate fetal biometry) or reduce stillbirth risk (some combination of sonography/biophysical profile or cardiotocography/non-stress test), each of which has established time frames for negative predictive value.

Of the five infants that survived in the setting of pregnancy-associated H5N1 in the 2025 review article by Purcell et al., four births were premature (<37 weeks) and were complicated by either spontaneous labor or need for emergent cesarean [10]. Regarding labor management, lessons from other pandemics would suggest that cesarean delivery should still be reserved for the most severe of cases, given the considerations of ventilation during cesarean birth. Other peripartum outcomes associated with cesarean include hemorrhage, surgical site infection or sepsis, and venous thromboembolism [27]. In the event of increasing work of breathing during labor or sepsis, careful consideration should be made regarding the anticipated length of labor when considering intrapartum cesarean for maternal indication on the grounds of worsening infection. In the event of the patient reaching full dilation and ensuring all typical obstetric criteria are met, operative vaginal delivery (OVD) may be considered in the event of substantial respiratory efforts during pushing to shorten the second stage of labor to avoid the potential morbidities of intrapartum cesarean. The recent COVID-19 pandemic provided data on effective positioning for prone ventilation of intubated gravidas for the most severe cases of acute respiratory distress syndrome (ARDS).

In the H1N1 2009 pandemic, early administration of antiviral neuraminidase inhibitor oseltamivir to pregnant individuals (<2 days onset of symptoms) was associated with a 4-fold lower risk of intensive care unit admission or death compared to delayed administration [28]. H5N1 shows some response to neuraminidase inhibition, though certain clades have high degrees of resistance [11]. Rapid test viral assays were frequently unreliable or false-negative during the H1N1 pandemic in 2009, potentially leading to a delay in therapy [28]. This suggests that clinical suspicion of disease should warrant early antiviral therapy among pregnant women. Tests may include antigen testing, which is faster but with lower sensitivity, or molecular assays with higher sensitivity but may take longer to result than point-of-care tests [29]. If patients report suspected flu symptoms and are pregnant, antivirals should be prescribed quickly. If an alternative etiology is diagnosed (example, COVID-19 or adenovirus) and flu testing is negative, then flu treatment may be discontinued. However, with high pre-test probability, patients should continue antivirals even when rapid testing is negative. Prophylactic antibiotics are not recommended. Patients should be advised that inability to tolerate oral hydration, rapid breathing or difficult breathing, unrelenting fevers, or new obstetric concerns (such as decreased fetal movement) should prompt evaluation in a clinical care setting while minimizing exposure to unaffected individuals.

For those patients with fever (100.4 F), there have been associations between untreated hyperthermia in pregnancy and certain birth defects, with some evidence that treatment of fever may help lower these risks [30]. Therefore, early treatment of febrile illness with antipyretics, such as acetaminophen, is advisable. It should be noted that despite some public discourse about association studies of acetaminophen and autism spectrum disorder, no high-quality studies have suggested causation [31] and international obstetric expert societies, such as the American College of Obstetricians and Gynecologists and the Society for Maternal-Fetal Medicine, continue to support the safety of this medication in pregnancy [32]. Seasonal influenza vaccination should continue to be recommended to all pregnant persons and persons planning to become pregnant, given the well-documented benefits of vaccine protection from existing seasonal strains [33].

## 5. Perinatal and Neonatal Pandemic Preparedness Planning

Attention should be given to preparedness planning for births in which the mother has an acute infection, and steps should be taken to minimize the risk of perinatal transmission to at-risk infants (Table 1). The CDC recommends that, if possible and desired by the family, the infant should be separated from the infected mother and cared for by a healthy caregiver [34]. Breastfeeding mothers should be encouraged to express their milk (after washing hands well with soap and water and with a hospital-grade breast pump to establish an appropriate milk supply). If the infant must room-in with the mother, or the mother desires co-location, hospitals can utilize physical measures to reduce the infant’s exposure, including physical barriers such as curtain between the mother and infant, keeping the infant at least six feet away, having another caregiver provide newborn care as much as possible, and having the mother wear a mask and practice hand hygiene when in close contact with the baby [34].

If the newborn is being cared for in a nursery or separate room by a healthy caregiver, standard precautions can be used. Symptomatic mothers and other visitors should not enter the nursery. The infant can be fed the mother’s expressed breastmilk, even if the mother is receiving treatment for influenza. If a newborn develops signs of possible illness, they should be examined by a provider and tested for influenza; infants who test positive should be considered for treatment with oseltamivir and placed on droplet precautions. Infants with influenza can continue to be breastfed, and, if unable to feed at the breast, can be fed expressed breast milk from a cup, syringe, or bottle [35].

After hospital discharge, symptomatic family members should be advised to continue to isolate and practice good hand and respiratory hygiene (e.g., wear a mask) to minimize exposure to the baby. Healthy caregivers should continue to care for the infant as much as possible until the mother is no longer symptomatic. All household members and close contacts of the newborn who are six months of age and older should receive the influenza vaccine [34].

## 6. Vaccine Strategy for Pregnant Individuals: Policy Recommendations and Research Gaps

Prioritizing available vaccines and access to vaccination for populations at increased risk for severe disease and poor health outcomes associated with flu, including pregnant persons, is a cornerstone of pandemic preparedness and response plans. All persons who are or will be pregnant during the annual influenza season in the US are currently recommended for vaccination in every pregnancy, timing vaccine receipt such that the duration of protection covers as much of the mother’s third trimester and/or birth up to six months of life for the infant during the typical flu season [36,37]. According to the 2020 update to the US framework for allocating and targeting pandemic flu vaccine in the event of a declared pandemic, pregnant persons at any stage of pregnancy are among the 26 million estimated US population that would be eligible for the earliest access to the vaccine [38]. Additional comorbid conditions may also impact individual risk among pregnant persons, including underlying respiratory or cardiovascular disease, diabetes, obesity, or compromised immunity. In situations of limited supply, which is to be expected at the start of a declared avian flu pandemic event, pregnant persons remain among the highest priority vaccination groups because of the high value placed on protecting both the mother and the fetus or infant up to 6 months of age with a single vaccination series. This same prioritization framework was successfully applied by the US during the only other flu pandemic of the 21st century that took place in 2009.

As of 2025, three pandemic flu AH5N1-monovalent vaccines with prototype influenza strains are currently licensed for use in humans by the US Food and Drug Administration (FDA), and several others are authorized by other regulatory bodies in Asia, Australia, and Europe [39]. Many countries, including the US, stockpile licensed vaccines [40]. None are commercially available or recommended for use in the US at this time, given that the risk of human infection remains low for much of the population. During a declared pandemic, these vaccines or other candidates currently under evaluation would be modified to target the circulating strain and be reviewed by regulatory bodies under rapid regulatory approval pathways like those that took place during the declared pandemics associated with the circulation of the 2009 H1N1 virus and the 2019 novel Coronavirus [41,42]. All three vaccines authorized for use in the US are inactivated influenza virus vaccines using egg or cell culture-based technology. There are many political and technical challenges in adequately preparing for a future H5N1 pandemic [43]. No currently mass-produced vaccines are currently available for the current highly pathogenic avian influenza H5N1 modern clades; however, vaccines have been stockpiled by some countries, including the FDA-approved AUDENZ™ (CSL Seqirus) M59^®^ adjuvanted, cell-based vaccine for individuals over the age of six months. The vaccine was not studied during pregnancy. There have been promising safety profiles for M59^®^ adjuvant in pregnancy without reports of teratogenicity [44]. Recently, human isolates from clade 2.3.4.4b H5N1 were successfully used to develop an mRNA vaccine candidate with promising results in a ferret model [45]. There are promising, rapidly scalable platforms in current use or development to suggest the hope for rapid development of a human candidate vaccine in the event of a future AIV pandemic. Meanwhile, the CDC tracks candidate vaccine virus (CVV) strains for future vaccine mass-production, which, depending on the vaccine platform, may be implemented quickly on an industrial scale using currently existing platforms.

Safety profiles for all H5 vaccines in the general population are favorable, based on an accumulation of data from clinical studies and extrapolating data from real-world experience with previous H1N1 products [39,42]. Other vaccine platforms that would not suffer from egg shortages during a bird flu pandemic are also being pursued; specifically, mRNA vaccines with candidates in early-phase clinical trials [46]. To date, however, pregnant individuals are systematically excluded from vaccine clinical trials, in line with standard clinical trial conduct practices, and therefore, data insufficiency remains a major impediment to having readily available safety data to inform recommendations and public acceptance regarding vaccination during pregnancy.

In a pandemic context, it is all but certain that vaccination during pregnancy will be recommended and supported by the leading obstetric and perinatal academic and professional societies in the US. The extent to which uncertainty must be navigated regarding the underlying evidence base for use of H5N1 pandemic vaccines in the pregnant population, both by healthcare workers and patients, is unknown, but there most certainly will be gaps in knowledge that will drive the research agenda during the post-commercialization and real-world implementation phases. There already is an abundance of real-world data confirming the safety of seasonal influenza maternal vaccination, and similar approaches to monitoring safety in real-time will be needed for a pandemic setting [47,48,49,50,51]. Some criteria in the evidence-to-recommendation framework used by the US CDC’s Advisory Committee on Immunization Practices (ACIP), namely cost-effectiveness, were abandoned during the COVID-19 public health emergency, while others were heavily emphasized, such as rapid and direct comparison of vaccination benefits to vaccination harms using simple modeling frameworks that were updated in real time as new safety and effectiveness data became available [52,53]. For both the public and healthcare providers, the implementation of a robust monitoring and evaluation framework to study and summarize the benefit-risk tradeoffs of H5N1 pandemic flu vaccination in pregnant persons will be crucial for acceptance due to the unique considerations that drive vaccine hesitancy and acceptance of maternal vaccination [54]. Further, ACIP has increasingly relied on a shared clinical decision-making style of recommendation or, at the very least, emphasized the role of healthcare provider counseling in making vaccine recommendations to patients. It will be critical that public health authorities make explicit recommendations and broadly disseminate the known benefits and risks, both to the mother and infant, of both vaccination and naturally acquired disease, to help support patient-provider conversations [55].

## 7. Conclusions

While there is limited data on H5N1 or novel avian influenza viruses’ effects during pregnancy, the available data suggest we should prepare for and anticipate cases of severe disease in the perinatal setting in the event of a future global pandemic. We outlined here the current epidemiology, unique pathophysiology of the disease in the setting of pregnancy, suggested clinical approach during pregnancy and birth, and suggested future directions regarding vaccine research and policy approaches to vaccination strategy. As new viral strains evolve, so should the approach in pregnancy management and close surveillance that will help mitigate risk and improve perinatal outcomes.

## Figures and Tables

**Table 1:** Proposed initial newborn care for H5N1 in pregnancy.

Rooming Considerations	Infant separated from mother and cared for by a healthy caregiver (preferred) OR Co-locate but use physical measures to reduce the infant’s exposure Curtain between the mother and infant Keep the infant at least six feet away from infected persons Another caregiver provides newborn care as much as possible Mother wears a mask and practices hand hygiene
Newborns with signs of illness	Provider examination Test and treat with oseltamivir if positive Continue to breastfeed, or, if unable, feed expressed breastmilk
After Discharge	Prioritize caregiving by healthy caregivers Symptomatic household members should isolate, wear a mask, and practice good hand hygiene All close contacts should receive the influenza vaccine when able

## Data Availability

No original data were presented in this article.
